# In Vitro Hepatotoxic and Neurotoxic Effects of Titanium and Cerium Dioxide Nanoparticles, Arsenic and Mercury Co-Exposure

**DOI:** 10.3390/ijms23052737

**Published:** 2022-03-01

**Authors:** Fernanda Rosário, Carla Costa, Cláudia B. Lopes, Ana C. Estrada, Daniela S. Tavares, Eduarda Pereira, João Paulo Teixeira, Ana Teresa Reis

**Affiliations:** 1 EPIUnit—Instituto de Saúde Pública, Universidade do Porto, 4050-600 Porto, Portugal; fe.8rosario@gmail.com (F.R.); joao.teixeira@insa.min-saude.pt (J.P.T.); ana.reis@insa.min-saude.pt (A.T.R.); 2 Laboratório para a Investigação Integrativa e Translacional em Saúde Populacional (ITR), 4050-600 Porto, Portugal; 3 Department of Environmental Health, Portuguese National Institute of Health, Rua Alexandre Herculano 321, 4000-055 Porto, Portugal; 4 Department of Chemistry and Aveiro Institute of Materials (CICECO), University of Aveiro, Campus de Santiago, 3810-193 Aveiro, Portugal; claudia.b.lopes@ua.pt (C.B.L.); ana.estrada@ua.pt (A.C.E.); danielatavares@ua.pt (D.S.T.); 5 Department of Chemistry and Center of Environmental and Marine Studies (CESAM), University of Aveiro, Campus de Santiago, 3810-193 Aveiro, Portugal; 6 LAQV-REQUIMTE—Associated Laboratory for Green Chemistry, University of Aveiro, 3810-193 Aveiro, Portugal; eduper@ua.pt; 7 CIIMAR—Interdisciplinary Centre of Marine and Environmental Research, University of Porto, Terminal de Cruzeiros do Porto de Leixões, Avenida General Norton de Matos S/N, 4450-208 Matosinhos, Portugal

**Keywords:** co-exposure, mixtures, HepG2, SH-SY5Y arsenic, mercury, titanium dioxide nanoparticles, cerium oxide nanoparticles, cytotoxicity, cell-cycle

## Abstract

Considering the increasing emergence of new contaminants, such as nanomaterials, mixing with legacy contaminants, including metal(loid)s, it becomes imperative to understand the toxic profile resulting from these interactions. This work aimed at assessing and comparing the individual and combined hepatotoxic and neurotoxic potential of titanium dioxide nanoparticles (TiO_2_NPs 0.75–75 mg/L), cerium oxide nanoparticles (CeO_2_NPs 0.075–10 μg/L), arsenic (As 0.01–2.5 mg/L), and mercury (Hg 0.5–100 mg/L) on human hepatoma (HepG2) and neuroblastoma (SH-SY5Y) cells. Viability was assessed through WST-1 (24 h) and clonogenic (7 days) assays and it was affected in a dose-, time- and cell-dependent manner. Higher concentrations caused greater toxicity, while prolonged exposure caused inhibition of cell proliferation, even at low concentrations, for both cell lines. Cell cycle progression, explored by flow cytometry 24 h post-exposure, revealed that TiO_2_NPs, As and Hg but not CeO_2_NPs, changed the profiles of SH-SY5Y and HepG2 cells in a dose-dependent manner, and that the cell cycle was, overall, more affected by exposure to mixtures. Exposure to binary mixtures revealed either potentiation or antagonistic effects depending on the composition, cell type and time of exposure. These findings prove that joint toxicity of contaminants cannot be disregarded and must be further explored.

## 1. Introduction

The dramatic growth of nanoparticles (NPs) production and the benefits they have to offer come with questions regarding the risks related to their exposure [1]. After being generated, NPs are released into the environment, affecting both biotic and abiotic components of the ecosystem and inevitably mix and interact with other contaminants, including metal(loid) and their compounds [2,3]. Consequently, these interactions raise concerns regarding their general and occupational health and safety profiles. Different NPs have been reported to have outstanding capacity for metals adsorption from aqueous or organic solutions [4,5,6,7,8], among them, cerium oxide nanoparticles (CeO_2_NPs) and titanium dioxide (TiO_2_NPs) nanoparticles. TiO_2_NPs hold immense potential, such as chemical stability and photocatalytic efficiency, and are commonly used in a variety of fields, from medicine and pharmacology to bioremediation [9,10]. TiO_2_NPs are also a main component of many household items [11]. The oxide form of the rare-earth metal cerium (CeO_2_) has antioxidant properties and due to that particularity, CeO_2_ particles are used in drugs for the treatment of medical disorders [12]. Moreover, CeO_2_NPs are present in outdoor air, since most of CeO_2_NPs applications benefit from its catalyst activity, including automobile catalytic converters and automotive fuel additives [13,14].

Arsenic (As) is a metalloid, and ubiquitous contaminant of natural environments. Chronic As exposure can cause cancer, neuropathies, bronchopulmonary and cardiovascular diseases, and chromosome aberrations [15]. The World Health Organization (WHO) established a provisional guideline value of 10 μg/L of As in drinking water [16], but in different parts of the world, As concentrations significantly higher than 50 μg/L have been detected [17]. Mercury (Hg) is another chemical that has received significant attention due to its neurotoxicity, long-range transport ability, volatility, persistence, and bioaccumulation in the environment and organisms. It has a lifetime of 1–2 years in the atmosphere and can be transported over long distances causing global mercury contamination [18,19]. Hence, some efforts have been made to develop effective pollution control technologies towards the efficient and enhanced As and Hg removal from contaminated sites. For instance, Zhou et al. [20] and Wang et al. [21], have successfully investigated mercury removal activities by photocatalytic oxidation and adsorption to CeO_2_−TiO_2_ nanoparticles and titania nanotubes (TNTs) for industrial application. Despite the positive outcomes from the interaction of NPs and legacy contaminants in environmental remediation, some studies have reported adverse effects on aquatic systems, soils and sediments resulting from mixtures [22,23,24,25]. For example, previous studies showed that the presence of TiO_2_NPs significantly enhanced the bioaccumulation and toxicity of Pb and As in zebrafish (Danio rerio), carp (Cyprinus carpio) and Daphnia magna [26,27,28].

Metal-nano adsorption may be an important process in mediating both NPs and metal(loid) toxic effects [29], as it could affect their reactivity and the way these contaminants interact with the cells [30] Nevertheless, the study of the adsorption behaviour has been largely disregarded in the toxicology context, and data on the adsorbed metallic contaminants to the NPs in the exposure medium are critically missing.

Human exposure to NPs is inevitable, and occurs through a variety of mechanisms, which include oral ingestion, inhalation and dermal penetration [31,32]. Due to their small size, large specific surface area and surface reactivity, these NPs can cross biological barriers, such as the blood-brain and blood-tissue barriers, translocate to the bloodstream and accumulate within the brain, liver, spleen and kidneys [33,34]. Different in vivo studies have investigated the TiO_2_NPs biodistribution and demonstrated that these NPs can reach the brain where they can lead to cell death and disturb brain functions, or even induce neurodegenerative disease and psychiatric disorders [35,36,37]. However, to the best of our knowledge, until now, there are no studies reporting the toxic potential of co-exposures to neuronal cells. Dosimetric studies identified the liver as the most sensitive target organ following inhalation exposure, and as such it serves as the critical target organ for setting an occupational exposure standard for airborne silver nanoparticles [38].

Although very limited data is found in the literature for in vitro studies on combined exposures, Wang et al. [39]. Found a synergistic genotoxic response in mammalian cells when exposed to TiO_2_NPs and As. It was also documented that the co-exposure to silver nanoparticles (AgNP) and cadmium (Cd2+) induced, in general, more toxic responses than individual exposures to AgNP or Cd2+. Foremost, co-exposure to AgNP and metals potentiated cell death by necrosis [40]. In our previous study on NP-metal co-exposure in A549 lung epithelial cells [41] we showed that As toxicity was reduced upon co-exposure with CeO_2_NPs or TiO_2_NPs, but the same was not observed with Hg, regardless of the concentration of NPs used, since we registered reduced mitochondrial activity and completely inhibition of cell proliferation, as observed when cells were exposed to Hg alone. These observations demonstrate that the toxic response to chemical mixtures is variable and that there is a need to address interactions of NPs in the context of mixed exposure towards human health. Considering the reality that NPs and widespread contaminants coexist in natural environments, and the lack of available data addressing its effects on mammalian cells, this study follows-up our previous paper [41] on the cytotoxic effects of TiO_2_NPs, CeO_2_NPs, As and Hg co-exposure to A549 cells.

In the present work we investigated the biological effects of single and combined exposures to TiO_2_NPs, CeO_2_NPs, As, and Hg in human hepatoma (HepG2) and human neuroblastoma (SH-SY5Y) cells through the WST-1 and clonogenic assays, and flow cytometry, to assess viability, proliferation, and cell cycle alterations, respectively. The NP dissolution and potential adsorption of the metals to the NPs in cell culture media was studied and considered in the interpretation of results. Ultimately, this work will contribute to the knowledge on the human impact of NP and metallic contaminants co-exposure.

## 2. Results

### 2.1. NPs Characterization

The overall results of TiO_2_NP and CeO_2_NP characterization are presented in Rosário et al., and a general characterization of the materials is provided in Figures S1–S3 of Supplementary Material.

Because the SH-SY5Y cell line required a different culture media, hydrodynamic size (Dh) and PdI were additionally determined in EMEM/F12K. In this (complete) culture medium, TiO_2_NPs Dh increased from 41.58 ± 0.04 to 115.73 ± 0.67 nm, and PdI decreased from 0.579 ± 0.01 and 0.181 ± 0.013 with increasing concentration (0.75 mg/L to 75 mg/L), at 0 h (Figure S3). Concerning CeO_2_NP, the Dh slightly decreased from 23.77 ± 0.11 at 0.075 µg/L to 19.30 ± 0.19 at 10 µg/L, while PdI was 0.468 ± 0002 and 0.428 ± 0.011, at the previously mentioned concentrations (Figure S3).

### 2.2. NP-Metal Adsorption and NP Dissolution

Possible adsorption of As and Hg to the nanomaterials was investigated in both culture media used in this work. The general results show a higher affinity of both metals for TiO_2_NPs than for CeO_2_NPs, which may be related to the concentration of the nanomaterials used, as the former was significantly larger than the latter.

**Figure 1:**
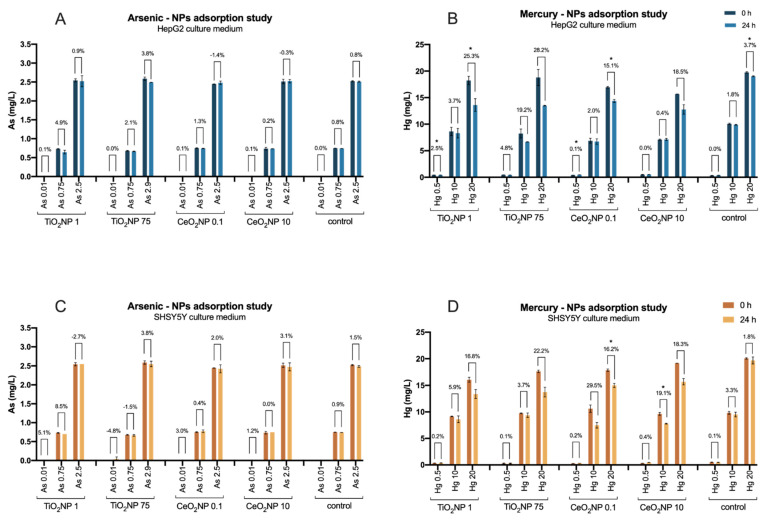
Adsorption of As (mg/L; (**A**,**C**)) and Hg (mg/L; (**B**,**D**)) to TiO_2_ (mg/L) and CeO_2_ (µg/L) in HepG2 (**A**,**B**) and SH-SY5Y (**C**,**D**) complete media, at 0 and 24 h, at all tested concentrations and mixture combinations. Controls (As or Hg without NP) are presented as well. Bars represent the concentration of the metal (mean ± standard deviation; *n* = 3) and the percentage of difference between concentration at the two time points is indicated. Statistical difference (paired *t*-test; *p* < 0.05) is indicated by *.

Adsorption of As to the nanomaterials was minimal, with a 5% maximum observed in the combination TiO_2_NPs 1—As 0.75 mg/L (Figure 1A,C). Adsorption to CeO_2_NPs was practically non-existent. In contrast, both nanomaterials, but particularly TiO_2_NPs, were more effective at adsorbing Hg from solution, in some cases reaching statistically significant differences between the concentrations at the two time points, as indicated in Figure 1. The percentage of Hg adsorption increased with the nanomaterial and metal concentration, varying between 2.5 and 28.2% in the presence of TiO_2_NPs, and 2 and 18% when CeO_2_NPs were used (Figure 1B,D). It should be noted that, in the literature, larger percentages of adsorption of metals to nanomaterials can be found [42,43,44,45]; however, these studies were made in water [46], while, in this case, the complex composition of the culture media (salts, proteins, lipids, glucose, serum, etc.) may interfere with the behavior of NPs, aggregation phenomena, and adsorption capacity.

Dissolution of Ti and Ce from the nanomaterials was not observed, as the concentration of the free ions remained unaltered after 24 h shaking, either when alone or mixed with As and Hg. Titanium concentration in solution was, on average, 0.59 and 1.28 mg/L at TiO_2_NPs 1 and 75 mg/L, respectively; cerium concentration was below the quantification limit.

### 2.3. Cell Viability

#### 2.3.1. Single Exposure

**Figure 2:**
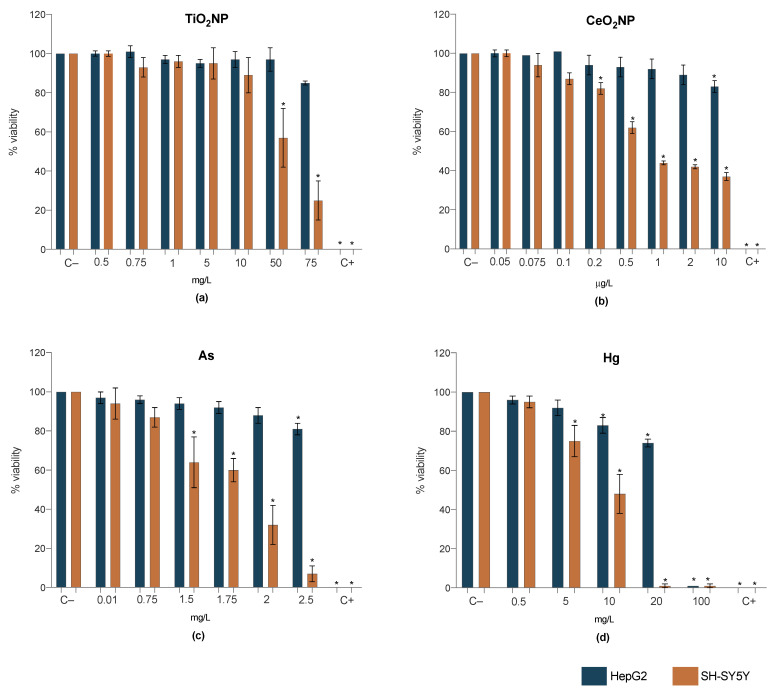
Results of cell viability from WST-1 assay for HepG2 (blue bars) and SH-SY5Y (orange bars) cell lines exposed for 24 h to TiO_2_NP (**a**), CeO_2_NP (**b**), As (**c**), Hg (**d**). C−: negative control; C+: positive control (Triton X-100 1%). Values are expressed as mean ± standard deviation (*n* = 4, each experiment in triplicate). * means significant differences between samples compared to C− (One-way ANOVA) at *p* < 0.05.

Figure 2 shows the cell viability of HepG2 and SH-SY5Y after 24 h of singles’ exposure, measured by the WST-1 assay. In more detail, TiO_2_NPs (1 and 75 mg/L), CeO_2_NPs (0.1 and 10 µg/L), As (0.01, 0.75 and 2.5 mg/L) and Hg (0.5, 10 and 20 mg/L) induced cytotoxicity to the HepG2 cell line only for the highest concentrations (Figure 2). Comparatively, SH-SY5Y cell line was more sensitive to all treatments, with a mitochondrial activity decrease in a dose-response manner. Mercury data revealed more severe toxic effects than TiO_2_NPs, CeO_2_NPs or As, with an abrupt decrease in viability at 10 mg/L (Figure 2d), something that was not observed in the HepG2 cell line.

#### 2.3.2. Co-Exposure

**Figure 3:**
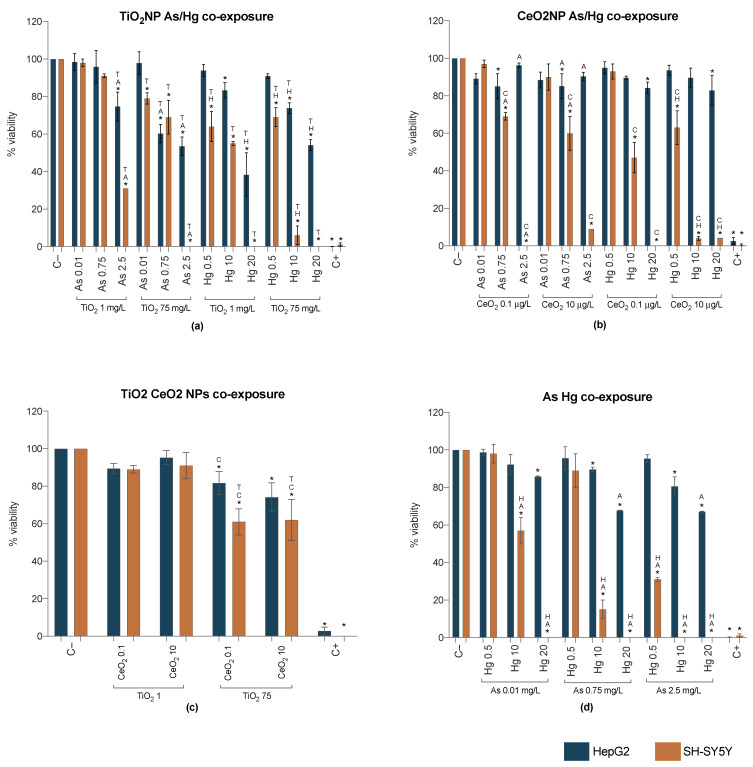
Cell viability from WST-1 assay for HepG2 (blue bars) and SH-SY5Y (orange bars) cell lines 24 h post-exposure to binary mixtures of NP and metals—TiO_2_NP-As/Hg (**a**), CeO_2_NP-As/Hg (**b**), TiO_2_NP-CeO_2_NP (**c**), As-Hg (**d**). C−: negative control; C+: positive control (Triton X-100 1%). Values are expressed as mean ± standard deviation (*n* = 4, each experiment in triplicate). * means significant differences between samples compared to C−; different letters (Titanium T; Cerium C) and/or metal (Arsenic A; Mercury H) indicate statistical difference from mixtures when compared to the compound alone (One-way ANOVA) at *p* < 0.05.

The cytotoxicity induced by TiO_2_NPs-As and TiO_2_NPs-Hg mixtures is explored in Figure 3a. In general, results for HepG2 indicate that, compared to single exposures, there is a potentiation of the effect when cells are simultaneously exposed to TiO_2_NPs and metals, inducing higher cytotoxicity in a concentration-dependent way. On the SH-SY5Y cell line, on one hand TiO_2_NPs 1 mg/L were able to attenuate the toxic effect of As, but not Hg. On the other hand, in the presence of As (0.01 and 0.75 mg/L) and Hg (0.5 mg/L), the cytotoxicity of TiO_2_NPs 75 mg/L was decreased; this antagonism was not observed for the remaining metals’ concentrations.

The co-exposure of CeO_2_NPs with As or Hg resulted in some interesting findings, as this NP (CeO_2_NPs 10 µg/L in SH-SY5Y; both concentrations in HepG2) was able to slightly attenuate the high toxicity of the metals, at their highest concentrations (As 2.5 and Hg 20 mg/L) but the opposite effect was observed for the lower concentrations of the metals. For example, Hg 10 mg/L when co-exposed with CeO_2_NPs 10 µg/L significantly lowered circa 10 times the mitochondrial activity of SH-SY5Y from 48% to 4%.

Compared to the single exposure, HepG2 treated with both NPs did not yield a markedly different response from the cells (Figure 3c). In contrast, an antagonistic effect was observed in SH-SY5Y, as the co-exposure to TiO_2_ and CeO_2_ NPs decreased the cytotoxicity they induced individually at the highest concentrations (Table S1). Still, a loss of circa 40% viability was observed.

Results from HepG2 cell line showed that the cytotoxicity from the co-exposure to As and Hg was concentration-dependent, with overall higher toxicity when compared to the corresponding single exposures (Figure 3d; Table S1). Once again, the neuronal cell line proved to be more sensitive, with several metals’ combinations causing near to 100% mortality.

### 2.4. Effects of 7 Days Single and Binary Exposures on Cell Proliferation

**Figure 4:**
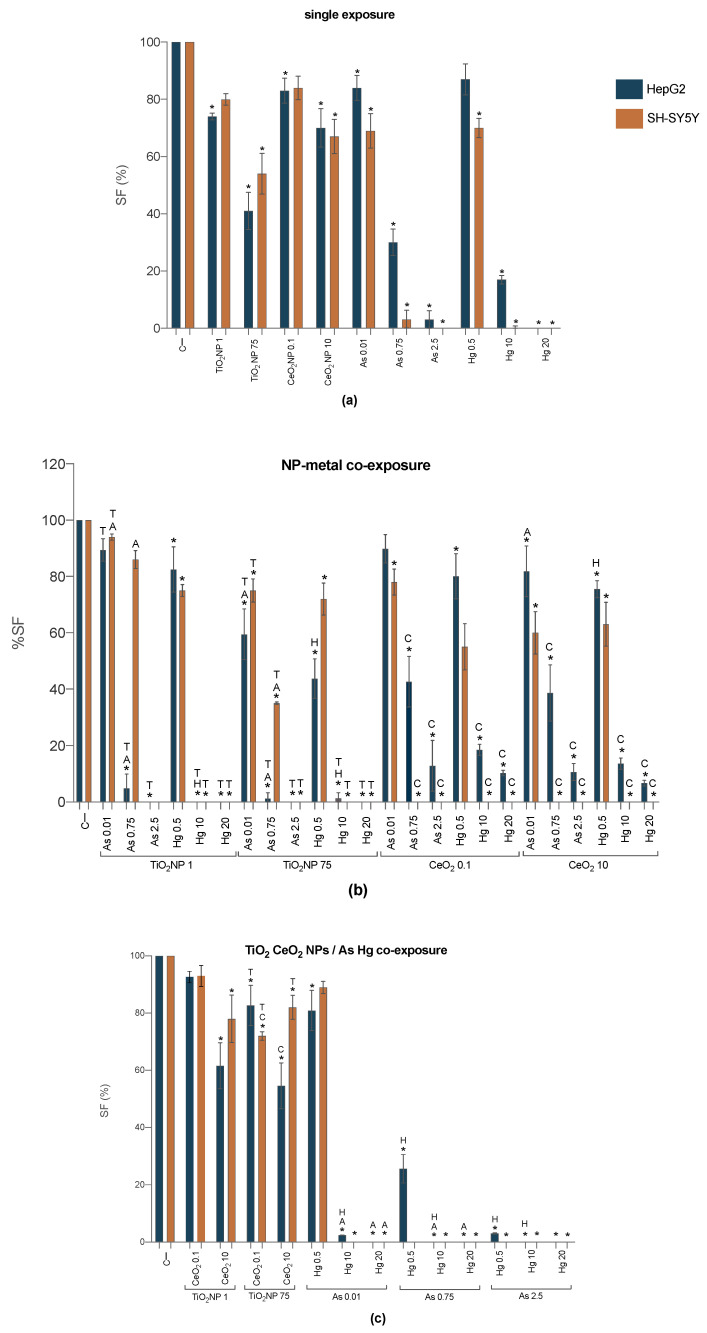
Surviving factor of HepG2 (blue bars) and SH-SY5Y (orange bars) cells 7 days post-exposure to single and binary mixtures of NP and metals—Single exposures (**a**), TiO_2_NP-As/Hg and CeO_2_NP-As/Hg (**b**), TiO_2_NP/CeO_2_NP and As/Hg (**c**). C−: negative control. Values are expressed as mean ± standard deviation (*n* = 3, each experiment in triplicate). * means significant differences between samples compared to C−; different letters (Titanium T; Cerium C) and/or metal (Arsenic A; Mercury H) indicate statistical difference from mixtures when compared to the compound alone (One-way ANOVA) at *p* < 0.05.

To investigate the proliferative capacity of cells under exposure to TiO_2_NPs, CeO_2_NPs, As and Hg for longer periods (7 days), the clonogenic assay was used (Figure 4; Table S2). For single exposures (Figure 4a), data showed concentration dependent significant inhibition of proliferation for all treatments and both cell lines. Arsenic and mercury proved to be more effective at hampering cell proliferation than the NPs, particularly at the highest concentrations (Hg 10 and 20 mg/L; As 0.75 and 2.5 mg/L), with the SF of SH-SY5Y decreased to zero. It should be mentioned that 7 days exposure to TiO_2_NPs caused a higher decrease in HepG2 cell survival than SH-SY5Y, indicating an inversion in cell sensitivity observed for 24 h exposure.

Longer exposures to TiO_2_NPs-As, TiO_2_NPs-Hg (Figure 4b) and As-Hg (Figure 4c) were significantly more toxic towards HepG2 cell line than the individual counterparts. With the exception of the lowest metal concentrations (As 0.01 and Hg 0.5 mg/L), no colony formation was observed for these mixtures, as the compounds seem to induce a long-term potentiation effect. The SF of SH-SY5Y increased when As was co-exposed with TiO_2_NPs (Figure 4b), even for the highest concentration, suggesting an antagonistic effect of TiO_2_NPs in As toxicity. However, TiO_2_NPs did not have the same protective effect on SH-SY5Y exposed to Hg, with evident decrease on the proliferative capacity especially for both TiO_2_NPs—Hg 10 mg/L and TiO_2_NPs 75—Hg 10 mg/L, when compared to control and the metal alone.

Compared to exposure to As or Hg alone, the SF of HepG2 cells was significantly increased by the presence of CeO_2_NPs suggesting an antagonistic or protective effect of this NPs, even if, when compared to negative control, the clonogenic data showed a significant decrease in SF. This protective effect, however, was not observed in SH-SY5Y.

The long-term effects induced by the mixture of the NPs (Figure 4c) do not present any pattern for any cell line, but overall, compared to single exposure, when the CeO_2_NPs were present the toxic effects of TiO_2_NPs were diminished. The highest toxicity for HepG2 was obtained for the combined exposure of TiO_2_NPs 75 mg/L and CeO_2_NPs 10 µg/L, while for SH-SY5Y was observed for TiO_2_NPs 75 mg/L and CeO_2_NPs 0.1 µg/L.

At last, under co-exposure to As and Hg, data showed a complete inhibition on SH-SY5Y colony formation (Figure 4c). One exception to this rule, was the metals’ mixture at their lowest concentrations, that seemed to induce a diminished toxicity than when alone. In HepG2 there is also an increased impairment of cell proliferation, particularly at higher concentrations of the compounds, where no colony formation was observed.

### 2.5. Cell Cycle Alterations

#### 2.5.1. Single Exposure

**Figure 5:**
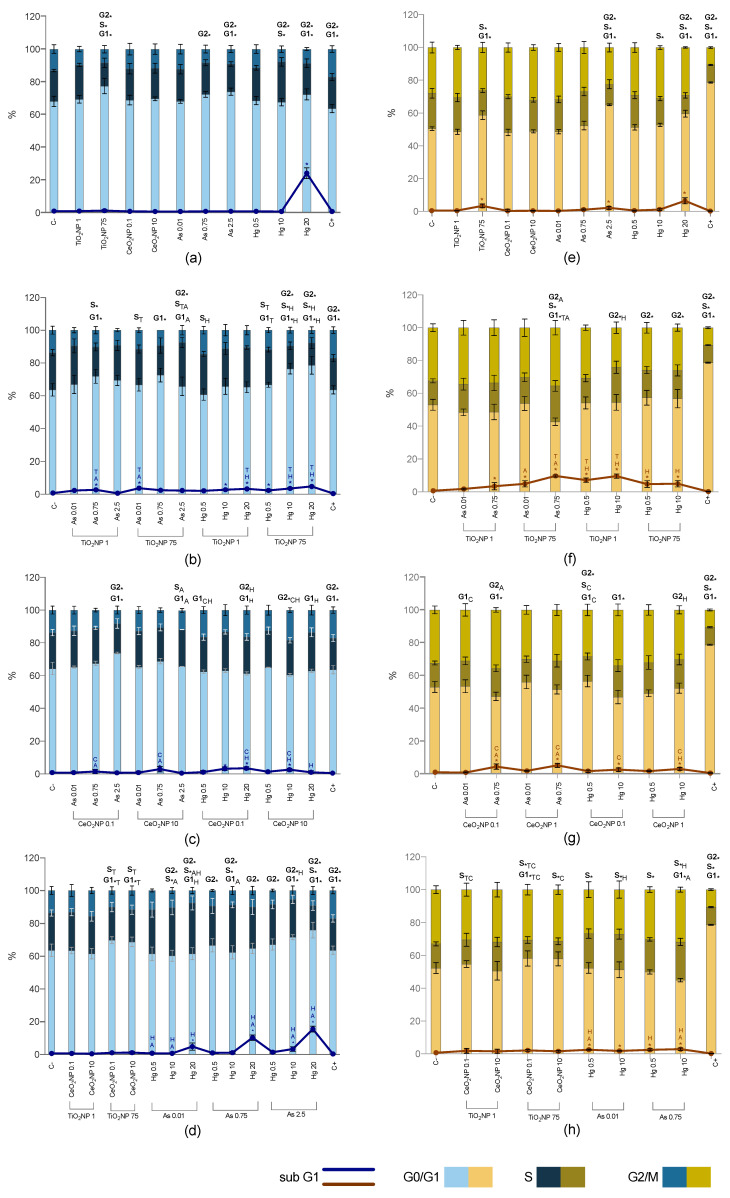
Cell cycle results of HepG2 (blue bars) and SH-SY5Y (orange bars) cell lines exposed for 24 h to single compounds (**a**,**e**), TiO_2_NP-As/Hg (**b**,**f**), CeO_2_NP-As/Hg (**c**,**g**), TiO_2_NP/CeO_2_NP and As/Hg (**d**,**h**). C-: negative control; C+: positive control (H_2_O_2_ 100 mM). Values are expressed as mean ± standard deviation (*n* = 3, each experiment in triplicate). * means significant differences between samples compared to C-; different letters (Titanium T; Cerium C) and/or metal (Arsenic A; Mercury H) indicate statistical difference from mixtures when compared to the compound alone (One-way ANOVA) at *p* < 0.05.

Figure 5a shows the distribution of the cell cycle in the different samples of the two studied cell lines after single exposures. Data from the cell cycle analysis is also presented as percentages of cells in each phase of the cell cycle (Table S3 of Supplementary Materials).

Data for HepG2 shows that the highest concentration of TiO_2_NPs induced a significant increase in G0/G1-phase at the expense of S- and G2/M-phases. The same concentration of TiO_2_NPs significantly increased the G0/G1-phase and decreased the S subpopulation for SH-SY5Y cells. No effects were observed for CeO_2_NPs when compared to control, for both cell lines.

Arsenic induced cell cycle alterations at concentrations higher than 0.75 mg/L for HepG2, with a decreased G2/M-phase, along with an increase in G0/G1-phase when the concentration of As was increased to 2.5 mg/L. The latter significantly increased the G0/G1-phase at the expense of S- and G2/M-phases for SH-SY5Y.

Furthermore, Hg induced several statistically significant alterations in the cell cycle of both cell lines. The exposure of HepG2 cells to Hg 10 mg/L decreased G2/M and increased the S-phase, while Hg 20 mg/L an increase in the G0/G1-phase was observed instead, coupled with the decrease in G2/M-phase. Regarding SH-SY5Y, the exposure to Hg 10 mg/L decreased the S-phase, while Hg 20 mg/L, increased the number of cells in G0/G1- and G2/M-phases at the expense of S-phase. At last, the sub-G1 was significantly increased for the highest concentration of TiO_2_NPs, As and Hg for SH-SY5Y, while the only effect observed at the sub-G1 subpopulation of HepG2 cells were at Hg 20 mg/L, where this population largely increased.

#### 2.5.2. Co-Exposure

The mixture TiO_2_NPs-As induced a cell cycle arrest in HepG2 (Figure 5b) at the G0/G1-phase for both TiO_2_NPs 1 mg/L—As 0.75 mg/L and TiO_2_NPs 75 mg/L—As 0.75 mg/L; a significant increase of the S-phase was also observed in the first case, while TiO_2_NPs 75 mg/L—As 2.5 mg/L exposure caused a decrease in G2/M-phase. Additionally, some TiO_2_NPs-As mixtures changed the cell cycle distribution when compared to TiO_2_NPs or As exposure alone, namely an increased S-phase when TiO_2_NPs 75 mg/L were combined with As 0.01 and 2.5 mg/L; the latter also reduced the percentage of cells in the G0/G1-phase in relation to As 2.5 mg/L alone.

Concerning SH-SY5Y, not so many alterations were observed after TiO_2_NPs-As exposures, with only significant differences to report in TiO_2_NPs 75 mg/L—As 0.75 mg/L (Figure 5f), where a decreased subpopulation of cells at the resting phase G0/G1 compared to control, TiO_2_NPs and As alone was observed, in addition to a cell cycle arrest at the S- and G2/M phases, compared to control and As alone, respectively. Moreover, a relevant increase of sub-G1 subpopulation was observed in most combinations.

For HepG2 exposure to TiO_2_NPs-Hg, statistical differences either in comparison to the control and/or Hg alone were mostly observed when the highest concentration of TiO_2_NPs was used (Figure 5b), namely a concentration dependent cell cycle arrest for the resting phase G0/G1, and a decreased subpopulation of cells at S and G2/M phases for TiO_2_NPs 75 mg/L—Hg 10 and 20 mg/L co-exposures. Additionally, all combinations increased the number of cells at sub-G1, but TiO_2_NPs 75 mg/L were able to decrease the percentage of cells in sub-G1 caused by Hg 20 mg/L alone. The co-exposure of SH-SY5Y to TiO_2_NPs-Hg decreased the number of cells at G2/M-phase to all concentrations, except to the lowest concentration of both compounds (TiO_2_NPs 1 mg/L—Hg 0.5 mg/L) when compared to control. When compared to Hg and TiO_2_NPs exposures alone, the cell cycle alterations were significantly diminished (Figure 5f).

CeO_2_NPs-metal mixtures did not cause such marked alterations in the cell cycle (Figure 5c). In fact, when compared to single exposures, in the presence of CeO_2_NPs (specially the highest concentration), the number of cell cycle alterations of HepG2 and SH-SY5Y cells induced by As and Hg, markedly decreased. Still, some combinations induced cell cycle alteration for both cell lines.

Regarding HepG2 cell line, the mixture of As 2.5 mg/L in combination with CeO_2_NPs 0.1 µg/L resulted in an arrest at the G0/G1-phase along with a decrease of the percentage of cells in G2/M-phase, compared to control, while the combination with CeO_2_NPs 10 µg/L increase the S-phase and decreased the G0/G1-phase in comparison to As alone. Ceria NPs co-exposed with Hg caused an overall increase in the G2/M cell population.

The exposure of SH-SY5Y to CeO_2_NPs-metal mixtures induced a cell cycle arrest at G0/G1-phase for the highest concentrations of As and Hg compared to control (Figure 5g). Also, an increase of the percentage of cells at G2/M-phase was observed for CeO_2_NPs 0.1 µg/L—As 0.75 mg/L compared to As single exposure, while some CeO_2_NPs-Hg exposures, caused a decrease in this phase if compared to the corresponding single Hg exposure.

Binary exposure to CeO_2_NPs and TiO_2_NPs induced a cell cycle arrest when the highest concentration of TiO_2_NPs was used (TiO_2_NPs 75 mg/L—CeO_2_NPs 1 µg/L and TiO_2_NPs 75 mg/L—CeO_2_NPs 10 µg/L) for both cell lines (Figure 5d,h). For SH-SY5Y there was an arrest at S-phase, while the same mixture induced a distinct effect on HepG2 cell cycle, with an increased G0/G1 population, when compared to control. Compared to exposure to these compounds alone, the cell cycle dynamics were significantly altered for the mixture exposure, suggesting antagonistic effects of both CeO_2_NPs and TiO_2_NPs.

At last, the analysis of simultaneous exposure to both metals induced the major number of cell cycle alterations for both cell lines and is presented in Figure 5d,h. Compared to negative control, the cell cycle dynamics of HepG2 were affected by the co-exposure to As and Hg, except for As 0.01—Hg 0.5 mg/L. All concentrations significantly decreased the percentage of cells in the G2/M-phases. Although, the distribution of HepG2 cells among the different cycle subpopulations was changed depending on the concentrations that were used, i.e., As 0.01mg/L—Hg 10 and 20 mg/L co-exposures induced a cell cycle arrest at G0/G1-phase, while As 2.5 mg/L—Hg 10 and 20 mg/L co-exposures induced a cell cycle arrest at the S-phase. Regardless of the concentration of As when in the presence of Hg 20 mg/L there was an increase in sub-G1 cell population. The increase of sub-G1 phase had already been observed after single exposure to Hg 20 mg/L, but the presence of high As concentrations decreased the effect, especially when the lowest concentration of As was used.

SH-SY5Y were more affected after co-exposure to As and Hg. All combinations increased the percentage of cells in sub-G1 while decreasing the S-phase. Along with an arrest at the resting phase G0/G1 when the concentration of As and Hg was increased to 0.75 mg/L and 10 mg/L, respectively.

## 3. Discussion

The use of single nanomaterials/nanoparticles in nanotoxicity studies overlooks the interactions between contaminants that occur in the environment, where more complex exposure scenarios are observed; NPs are expected to interact with other contaminants, namely ubiquitous metals, such as As and Hg [2]. Current studies on joint toxicity of these compounds, especially on hepatotoxicity and neurotoxicity are limited. Thus, in this work we investigated the single and joint hepatotoxicity and neurotoxicity of NPs and metals. To our best knowledge, this was the first time to investigate these binary mixtures toxicity and behavior towards HepG2 and SH-SY5Y cell lines. In our previous study, it was demonstrated that combined exposures changed the lung toxicity in A549 cell line [41]. In the present study, in order to confirm the toxic potential of single vs. co-exposures, we carried out cell viability through the WST-1 test, which measures mitochondrial viability, and cell proliferation experiments, complemented by analysis of the cell cycle, a valuable tool to understand the mechanism underlying the cytotoxicity of some compounds. Most of the studies addressing NP co-exposure with other contaminants treat the “classic” contaminants as the primary analyte and subsequently investigate the influence of NPs on their toxicity [2]. In the current study we go further and also analyze the influence of the metal (or other NP) on the NPs toxicity. Hence, the co-exposure cytotoxicity and cell cycle alterations were explored for several combinations of the xenobiotics: NP-metal; NP-NP; and metal-metal.

### 3.1. Single Exposures

The results here presented show a different cytotoxic response of HepG2 and SH-SY5Y that has equally been observed by other authors for TiO_2_NPs [47], CeO_2_NPs [48], As and Hg [49], with the neural cell line being more sensitive than HepG2 in all cases, after 24 h exposure. The same authors also observed a dose-dependent cytotoxic effect as observed in this study. Moreover, a time-dependent effect was registered; shorter exposures were less cytotoxic than the longer ones.

Cell-cycle analysis of single exposures revealed that TiO_2_NPs, As and Hg but not CeO_2_NPs, changed the profiles of SH-SY5Y and HepG2 cells in a dose-dependent manner. In general, a distinct pattern of effects were obtained for HepG2 and SH-SY5Y. In HepG2, the most common observation is the arrest of cycle progression at the G0/G1 phase after TiO_2_NPs and As exposure, and a decrease in G2/M-phase for the highest concentration of all the compounds. The checkpoints are controls on cell division and are commonly viewed as critical points on the progression of the cell cycle and consequently on cell survival [50]. The cell cycle arrest of HepG2 at G0/G1 checkpoint/transition activates repair pathways and the cells may have an extra time to repair DNA damage prior to segregation of chromosomes, which explains the lower sensitivity of this cell line. For the SH-SY5Y cell line, the arrest at G0/G1 is accompanied by a decreased S-phase and increase in the number of events in the sub-G1 region, the latter particularly indicating that DNA damage could not be repaired and the cell entered apoptosis, this way justifying the decreased cell viability (WST-1 assay).

In both cell lines, metals induced more alterations in the cycle, which corroborates the higher toxicity of metals in relation to NPs, particularly the inhibition of cell proliferation at 7 days exposure. In fact, after 7 days of exposure to TiO_2_NPs and CeO_2_NPs SH-SY5Y showed a high-level resistance compared to 24h exposure, in a way that was similar to the proliferation results of HepG2 cell line. A large number of publications has reported that some metals and metalloids can disrupt important cellular mechanisms responsible for growth, proliferation, differentiation, damage-repairing processes, and apoptosis, as reviewed by Balali-Mood et al. It is also known that metal(loid)s, including As and Hg, have the ability to bind to regulatory proteins involved in cell cycle regulation [51]. Indeed, in this study arrests in all phases of the cycle were observed in a dose-depending manner (the higher the concentration, the more disruption of cycle phases was noted). As so, synthesis of proteins and cell cycle progression were compromised, causing retardation or inhibition of cell growth in longer exposure periods. Other mechanisms can be involved in the higher toxicity observed for metals. The marked increase of sub-G1 phase, particularly at elevated Hg concentrations, suggests DNA damage and/or impairment of DNA repair mechanisms, since sub-G1 population is usually associated with DNA fragmentation often resulting from the late stages of apoptosis or double-strand breaks in DNA [52,53]. There have been conflicting reports about the effects of metals on the cell cycle but the reduction of G0/G1 phase and S phase arrest by Hg has previously been reported in SH-SY5Y cells [54]. Since the cell-cycle of SH-SY5Y cells was more affected than HepG2, this can be the justification for the greater sensitivity of the neural cell line.

### 3.2. Arsenic-Mercury Co-Exposure

The reduction of cell viability after combined exposure to As and Hg followed the same trend as in single exposure, i.e., cytotoxicity was dependent on concentration, time of exposure and cell type. At 0.5 mg/L of Hg, viability was similar to the individual toxicity of As and Hg in both cells; increasing Hg concentrations resulted in increased cytotoxicity, more prominent in SH-SY5Y after 24 h exposure. A deviation to this pattern was observed when both cell lines were exposed to As 2.5 mg/L—Hg 0.5 mg/L, after which an antagonistic effect (in relation to As) was observed. Cell cycle analysis confirms that the distribution of cells among the different phases was practically not affected in this mixture. It is not the first time that As-Hg antagonism at specific concentrations is reported [55], indicating that more mechanistic studies are needed to further explore this matter. After 7 days exposure there was a visible potentiation effect of the mixture in both cell lines, as proliferation was markedly decreased and, in many combinations, reduced to zero. Hence, cytotoxicity resulting from long-term exposure to metal mixtures was greater than the one observed for the single metals alone.

A substantial increase of the sub-G1 phase was registered, which, as previously discussed, may be indicative of DNA lesions such as oxidative DNA damage, DNA strand breaks and DNA crosslinks [56,57,58]. It is, therefore, expected that, when both metals are present, there is an amplification of genotoxicity and consequently, cell death.

### 3.3. TiO_2_NPs—CeO_2_NPs Co-Exposure

Results show that simultaneous exposure of HepG2 to CeO_2_NPs and TiO_2_NPs are not significantly different from exposure to each NP alone, but the mixture yielded an antagonistic cytotoxicity in SH-SY5Y, which was more evident after prolonged exposure. This difference may be a result of the absence of co-aggregation behavior of NPs in culture media [59,60]. While in HepG2 culture media, TiO_2_NPs 75 mg/L—CeO_2_NPs 10 µg/L increased from 115 nm to 250 nm, in SH-SY5Y culture media the hydrodynamic size did not change by the addition of NPs (from 116 to 145 nm). Since CeO_2_NPs and TiO_2_NPs are both negatively charged and no co-aggregation behavior was observed, both could compete for the adsorption sites on the cell wall.

Despite the absence of data on prolonged exposure of binary mixtures in the literature, this result is in agreement with our previous study on A549 cell line [41]. Hence, data for CeO_2_NPs/TiO_2_NPs was similar for the three tested cell lines.

### 3.4. NP-Metal Co-Exposure

Simultaneous exposure to NPs and metals is complex and depends on the physical and chemical interaction between the NPs and the metal ions, the dose of each compound and time of exposure, aggregation phenomena, and cell sensitivity, among others. A number of mechanisms have been proposed to explain the interaction between NPs and metals. The capacity of metal oxide NPs to adsorb contaminants, followed by the uptake of the NPs-metal complexes, can play a decisive role in the toxicity of either contaminant [2,61,62], therefore addressing the NP-metal adsorption potential is an important consideration for the study of co-exposure effects. Our results show that adsorption of As and Hg to TiO_2_NPs and CeO_2_NPs was not substantial, hence it does not solely explain the toxicity alteration observed in co-exposures; regardless its influence in the outcome must not be disregarded.

Indeed, our experiments confirm the complexity of combined exposure to NPs and metals, as both potentiation and antagonism were observed, depending on the chemicals involved, their doses and the cell line. Also, toxicity may increase in relation to one of the components of the mixture while decrease in relation to the other, as will be explained in the following paragraphs.

Remarkably, CeO_2_NPs were able to reduce the individual toxicity of As and Hg at the highest concentrations, after 7 days exposure in HepG2, suggesting a cytoprotective effect, which has previously been reported by other authors [63,64,65]. A similar attenuation effect at 7 days exposure was observed when SH-SY5Y were exposed to mixtures of TiO_2_NPs and As 0.01 and 0.75 mg/L. These antagonistic effects were not, however, so noticeable after 24 h of co-exposure, suggesting that whatever phenomenon occurring that exerts cytoprotection upon metal co-exposure may require several cell cycles. Indeed, after 24 h exposure to these mixtures, changes on the cell cycle were not significant. One interesting finding concerning antagonism was the ability of low concentrations of metals (As 0.01mg/L and Hg 0.5mg/L) to reduce the toxicity of TiO_2_NPs and CeO_2_NPs on both HepG2 and SH-SY5Y, which may be explained by several mechanisms of physico-chemical and/or biochemical nature.

First, it can be explained by aggregation and sedimentation phenomena Aggregation of NPs is likely to occur in an elevated ionic strength environment, such a culture media, as confirmed by the increase of the hydrodynamic size of TiO_2_ and CeO_2_ NPs when compared to water [41] and, in turn, promote the sedimentation of the NPs. Additionally, the addition of metal(oid)s seems to increase aggregation to a greater degree, as otherwise observed by Wang et al. [66] and supported by our own DLS data, which shows that the hydrodynamic size of TiO_2_NPs and CeO_2_NPs increased in the presence of As and Hg, when compared to the NPs alone. For example, TiO_2_NPs 1—As 0.75 mg/L mixture has an average hydrodynamic size of 120 nm that increases to 260 nm in the TiO_2_NPs 75—As 0.75 mg/L mixture; TiO_2_NPs 1—Hg 10 and TiO_2_NPs 75 -Hg 10 mg/L have a respective size of 122 and 216 nm. In the first place, this increase in size will cause the diminishing of the superficial area and, therefore, decrease the cytotoxicity of the NPs (i.e., comparatively to the single NP). Decreased cell damage, as a function of the concentration, has already been reported in two previous studies that also observed that co-exposure of As with high concentrations of TiO_2_NPs increased the formation of aggregates and decreased the toxicity [39,67]. But also, the increased sedimentation of NPs can create a physical barrier and partially block or retardate the metal access to the cells, this effect being more likely when the metal(oid) concentration in solution is low and more evident after a longer exposure period. Indeed, our data demonstrate that this effect of metals in NP aggregation is even more complex, as additionally to being dependent on the concentration of the NP (size increases with increasing NP concentration), it is inversely dependent on metal concentration. Therefore, even if toxicity is decreased when compared to the NP alone, it may be increased when compared to the metal single exposure in a dose-dependent manner, as observed in HepG2 and SH-SY5Y cells, both after 24 h or 7 days exposure. Again, the analysis of the hydrodynamic size is important to understand these results. In the mixtures of highest concentration of As and Hg with TiO_2_NPs and CeO_2_NPs, a decrease in the hydrodynamic size was observed (e.g., TiO_2_NPs 75 mg/L with As 0.75 and 2.5 mg/L had 259.9 and 159.8 nm, respectively); the smaller aggregates facilitate the entrance of NP and metals (by decreasing deposition) in the cells. The entrance of NPs, along with the NP-metal complexes (even if adsorption was low, it occurred to some extent, particularly Hg to TiO_2_NPs), and with the toxicity of the higher concentration of the free ions (As and Hg), which was observed in the single exposure data, could be responsible for the potentiation of the toxicity from the mixtures NPs/metals, which increases with As or Hg concentration increase. Other authors observed that higher toxicity could be a result of numerous interactions’ mechanisms that can occur between NPs and other contaminants [2]. First, some contaminants can directly affect the physiological activities of organisms and change receptor tolerance to the NPs. Second, contaminants may disrupt the physical integrity of the cell membrane or increase the hydrophobicity, which subsequently could promote NPs internalization and toxicity. Third, Wang et al. [68] found that heavy metal ions can increase the toxicity of NPs by heightening the intracellular retention of NPs or damaging the cell membranes. Another mechanism shows that some contaminants, such as natural metals, can scavenge NPs-produced ROS to generate additional Reactive Oxygen Species (ROS) to increase toxicity, DNA damage and inhibiting DNA repair leading to apoptosis. Indeed, the shift towards the G0/G1 and sub-G1 phases in TiO_2_NPs-metal mixtures, indicators of apoptosis, support the observed increased loss of viability in both cell lines.

It should be noted that this last mechanism can also be responsible for the attenuation of the toxic effect often observed when CeO_2_NPs were present, in HepG2, and previously in A549 [41], as this nanomaterial has the ability to scavenge ROS and minimize oxidative stress [62,69].

One limitation of this study is that it does not account for possible biotransformation processes that the NPs may undergo once inside the organism (e.g., aggregation, dissolution in body fluids). Future work could address these possible changes and include characterization of the nanoparticles after absorption by the organism (e.g., TEM analysis after in vitro cell exposure; analysis of surface modification, agglomeration, colloid formation and stability in relevant biological fluids), to more realistically assess the potential nanotoxicity in internal organs. This, however, represents an analytical challenge, particularly in in vitro assessments that lack the complexity and metabolism of organisms that in vivo experiments provide.

## 4. Materials and Methods

### 4.1. Materials

Titanium dioxide NPs (CAS No. 13463-67-7; #700347; <150 nm—anatase/rutile ca. 80:20) and cerium oxide NPs (CAS No. 1306-38-3; #643009; <25 nm) were purchased from Sigma-Aldrich. Sodium meta-arsenite (NaAsO_2_, ≥90%, CAS No. 7784-465-56) and mercury (II) chloride (HgCl_2_; CAS No. 7487-94-7) were purchased from Sigma-Aldrich and Merck, respectively. Nitric (HNO_3_ 65%; CAS No. 7697-37-2) and fluoridric (HF 40%; CAS No. 7664-39-3) acids were both from Merck. H_2_O_2_ (CAS No. 7722-84-1) and Triton X-100 (CAS No. 9002-93-1) were bought from Sigma-Aldrich (St. Louis, MO, USA).

### 4.2. Nanoparticle Dispersion and Solutions

The stock solutions of NPs were prepared daily in sterile distilled water (dH_2_O) at final concentrations of TiO_2_NPs 200 mg/L and CeO_2_NPs 10 mg/L. The solutions were vortexed for 1 min, sonicated for 15 min in an ultrasonic bath (Bandelin Sonorex RK100, Berlin, Germany) to disperse NP agglomerates and aggregates, and vortexed again for another min. Nanoparticles were then serially diluted in complete culture medium to the desired concentrations according to the test to be performed and thoroughly vortex immediately before use. Solutions of arsenic and mercury were prepared daily in complete culture medium and vortexed before use.

### 4.3. Nanoparticle Characterization

TiO_2_NPs and CeO_2_NPs were characterized previously [41]. Additionally, as a different culture media is required for the growth of SH-SY5Y cells, the hydrodynamic diameter and polydispersity index values (PdI) of both NPs were measured using the same methods described in Rosário et al. For HepG2, the same complete media as A549 was used, therefore the characterization is the same as presented in our previous work [41].

### 4.4. NP-Metal Adsorption and NP Dissolution in Cell Culture Media

To check for possible adsorption of the metal(loid)s to the nanomaterials, complete culture media was spiked with As, Hg, TiO_2_ and CeO_2_ nanomaterials at all concentrations and mixtures’ combinations tested. The possible dissolution of the metallic nanomaterials was assessed in the presence and absence of As and Hg, by determining Ce and Ti concentration after nanomaterial removal.

The solutions were stirred in acid-washed borosilicate glass flasks, at 90 rpm, for 24 h, with continuous temperature and pressure monitoring. For each experiment, the concentration of the targeted contaminants in solution was measured at 0 h and 24 h. To remove the nanomaterials, 1.5 mL of solution were aspirated and centrifuged for 30 min, at 30,000× *g*, followed by a second centrifugation of 1 mL of the supernatant, at the same conditions. Three independent experiments of two replicates each were run for each mixture. To check for As or Hg losses, one flask of each metal(loid) concentration without the nanomaterials was kept under the same conditions for the entire procedure, per independent experiment. Additionally, one flask per batch was left only with the culture media to detect possible contaminations.

Prior to ICP-MS quantification, samples were acid digested (As; Ce: 500 uL of supernatant with 500 µL HNO_3_ 65%; Ti: 200 uL of supernatant with 500 µL HNO_3_ 65% and 24 uL of HF 40%), in a Thermoblock, at 60 °C for 18 h.

Blanks (culture media) were also digested to check for contamination during the digestion procedure. ICP-MS measurements were performed in a Quadrupole Thermo Scientific X Series ICP-MS, equipped with a Peltier Nebulizing Camera and a Burgener Nebulizer. Samples were pumped by 3-channel peristaltic pump, concentric glass nebulizer and glass cyclone spray chamber. Solutions for Hg analysis were acidified to pH < 2 with HNO_3_ 65% and kept at −20 °C until analysis by thermo desorption-atomic absorption spectroscopy (TD-AAS) with gold amalgamation, as described by Costley et al. [70] and Reis et al. [71] NRC TORT-3 certified reference material was used to check the equipment’s daily accuracy. Total Hg concentration was within the certified reference interval (Hg = 0.30 ± 0.01 mg/kg; *n* = 12) and the relative standard deviation (RSD) among replicates was <10%.

The percentage of adsorption was calculated according to Equation (1), after blank and control correction (if necessary).

% adsorption = (metal concentration_t_ = 0 _h_ − metal concentration_t_ = 24 _h_ ÷ metal concentration_t_ = 0 _h_) × 100 (1)

### 4.5. Cell Culture

Human hepatocellular carcinoma HepG2 (ECACC 85011430) and human neuroblastoma SH-SY5Y (ECACC 94030304) cells lines were obtained from the European Collection of Authenticated Cell Cultures (ECACC). The HepG2 cell line was cultured in complete growth medium (Dulbecco minimal Eagle’s medium (DMEM) with 1% antibiotic-antimycotic solution (100 units/mL of penicillin, 100 µg/mL of streptomycin) and 10% (*v*/*v*) fetal bovine serum (FBS)). Culture media of SH-SY5Y cell line, consisted of a nutrient mixture Eagle’s minimum essential medium (EMEM)/Kaighn’s Modification of Ham’s F-12 Medium (F12K) supplemented with 15% FBS, 1% NEAA (Non-Essential Amino Acids) and 1% antibiotic and antimycotic solution. Cells were grown at 37 °C, 5% CO_2_, in a humidified atmosphere. Cell confluence and morphology were observed under an inverted phase contrast microscope Nikon Eclipse TS100 (Japan) and subcultured when confluence reached 80% using 0.05% trypsin/1 mM EDTA or 0.25% trypsin/1 mM EDTA (GIBCO). Depending on the assay, cells were seeded in 96, or 6 well plates and left 24 h for adhesion. After that time, the culture medium was replaced with fresh medium containing single or binary mixtures of TiO_2_NPs, CeO_2_NPs, As, Hg solutions.

### 4.6. Exposure to Single or Binary Mixtures of TiO_2_NPs, CeO_2_NPs, as and Hg

Cells were exposed to TiO_2_NPs (0.5–75 mg/L; 0.15–20 µg/cm^2^), CeO_2_NPs (0.05–10 μg/L; 0.015–3 ng/cm^2^), As (0.01–2.5 mg/L), and Hg (0.05–100 mg/L). Human exposure to TiO_2_NPs comes from several sources, including, among other, environmental [72,73], food products, with an estimated consumption of 1 mg TiO_2_/kg bw/day in adults [74] and personal care products, through which it has been estimated that the range of exposure ranges from 0 to 10 mg TiO_2_ per day [75]. To our best knowledge, concentrations of human exposure to CeO_2_ NPs have not been reported; therefore, the lowest value of the tested range was based on current estimations of these NPs in the environment, which varies from 0.1 to 1.0 µg/L in the aquatic compartment [76], while values of 1.12 mg/kg have been reported in soil [77]. Benameur et al. [78] considered an environmentally relevant dose of CeO_2_ NPs to be 60 µg/L. Lowest concentrations of As and Hg correspond to the maximum allowable concentration for drinking water [79] and fish [80], the major sources of human exposure to these elements, respectively. The high end of the ranges corresponds to concentrations where a decrease in viability was observed for each contaminant.

For binary mixtures two concentrations of NP and three concentrations of metals were chosen based on results of single exposure: TiO_2_NPs (1 and 75 mg/L), CeO_2_NPs (0.1 and 10 μg/L), As (0.01, 0.75 and 2.5 mg/L) and Hg (0.05, 10 and 20 mg/L). To allow for adsorption equilibrium of metals to NPs, mixture solutions were prepared in complete culture media and left in an orbital shaker (90 rpm) for 24 h, at room temperature [4,81].

### 4.7. Cell Viability by WST-1 Assay

Cell relative viability was determined using the WST-1 (water-soluble tetrazolium) assay, after 24 h of exposure to single and binary mixtures. This assay measures mitochondrial metabolic activity of viable cells reducing 2-(4-iodophenyl)-3-(4-nitrophenyl)-5-(2,4-disulfophenyl)-2H-tetrazolium. HepG2 and SH-SY5Y cells were seeded on 96-well plates at a density of 3 × 105 cells per mL (100 μL). After 24 h, the culture medium was replaced by 100 μL of single or binary mixtures at desired concentrations of compounds and treated for 24 h. Culture medium was used as negative control, whereas Triton X-100 (1%) was used as positive control. Following incubation, 10 μL of WST-1 reagent was added to the samples which were incubated for further 2 h at normal culture conditions. Optical density (OD) was read on SpectraMax^®^ iD3 multi-mode microplate reader (Molecular Devices, San José, CA, USA) at 450 nm. Relative cell viability (%) was expressed as a percentage relative to untreated control cells. The interference of NPs and metals in the assays was eliminated by reading sample blanks (cell-free) for all the concentrations tested. All experiments were performed at least in triplicate on three separate occasions.

### 4.8. Cell Proliferation by Clonogenic Assay

The clonogenic cell survival assay was used for the determination of the ability of HepG2 and SH-SY5Y cells to proliferate after exposure to the test compounds and mixtures. The assay was performed as described by Franken et al. [82] with minor modifications. Cells were seeded at 200 cells/well in 6-well plates for 24 h and treated for 7 days with single or binary mixtures of CeO_2_NPs, TiO_2_NPs, As and Hg. Cells were then washed with PBS, fixed and stained with a mixture of 0.5% crystal violet in ethanol (Merck). Colonies with more than 30 cells were counted and the plating efficiency (PE) and surviving factor (SF) were calculated according to Equations (2) and (3), respectively. PE is the average of three independent counts of two replicates each. Culture medium was used as negative control in all experiments, and Triton X-100 (1%) was used as a positive control.

PE = number of colonies counted number of cells plated × 100(2)

SF = PE treatment/PE negative control × 100(3)

### 4.9. Cell Cycle Analysis—Flow Cytometry

Cell cycle analysis was performed according to Oliveira et al. [83] with some modifications [41]. Cells were seeded in 24-well plates at a density of 3 × 10^5^ cells/mL and exposed to 600 μL of single and binary mixtures of TiO_2_NPs, CeO_2_NPs, As, Hg. Culture medium was used as a negative control, and H_2_O_2_ 100 mM was used as positive control. After 24 h, cells were washed with PBS pH 7.2 (1X), trypsinized and centrifuged at 700× *g* for 5 min. The supernatant was discarded, and the cultures were resuspended in PBS pH 7.2, followed by centrifugation at 700× *g* for 5 min. Cells were then resuspended in cold 70% ethanol for fixation and stored at −20 °C until further analysis. At the time of analysis, cells were centrifuged at 700× *g* for 5 min and resuspended in cytometry grade PBS (pH 7.2). Then, 50 μg/mL of propidium iodide (PI) and 50 μg/mL of RNase were added to stain nuclear DNA and remove RNA from the samples, respectively. Samples were incubated for 15 min in the dark. Relative fluorescence intensity was measured in a Guava easyCyte 8HT Benchtop Flow Cytometer (Luminex, Austin, TX, USA). Acquisitions were made using Guava InCyte Software 3.1. For each sample, the number of events reached approximately 10,000. Debris and doublets were excluded by the definition of a specific region (side scatter-width vs. side scatter-area). Cell cycle analysis of the single population was then conducted based on the DNA histogram outputs by calculating the percentages of cells occupying the different phases of the cell cycle (G0/G1, S, and G2/M; Equation (4)). Additionally, the percentage of sub-G1 cells, usually associated with small DNA fragments, was estimated according to Equation (5), by the analysis of the broad hypodiploid peak below the G0/G1 phase. Three independent experiments consisting of three replicates each were performed.

% phase X = (number cells phase X ÷ sum cells phase G0/G1 + phase S + phase G2/M) × 100(4)

% phase subG1 = (number events phase subG1 ÷ sum events subG1 + cells phase G0/G1 + phase S + phase G2/M) × 100(5)

### 4.10. Statistical Analysis

The results are reported as means ± standard deviations (SD). Statistical differences between As and Hg concentrations at 0 and 24 h in adsorption experiments were evaluated by paired *t*-test in Prism 9 (GraphPad Software, San Diego, CA, USA). Remaining data analysis was performed in the software SigmaPlot version 11 (Systat Software Inc., San José, CA, USA), by one-way ANOVA analysis of variance (*p* < 0.05) followed by Holm-Sidak test or the Dunn’s test for the parametric and non-parametric data, respectively. The differences were considered statistically significant for *p* < 0.05. Graphs were designed in Prism 9 (GraphPad Software).

## 5. Conclusions

This work aimed at assessing the toxic potential of metal oxide nanoparticles (TiO_2_NPs and CeO_2_NPs) and metal(loid)s (As and Hg) individually and in co-exposure on HepG2 and SH-SY5Y cell lines, as a respective measure of hepatotoxicity and neurotoxicity. All single compounds were able to induce cytotoxicity, depending on the dose and time of exposure. Higher concentrations induced higher toxicity, and metals showed greater toxicity than NPs. SH-SY5Y cells were more sensitive than HepG2 cells.

The interpretation of the interaction between NPs and other contaminants is complex and requires a case-to-case analysis. Based on these results and from our previous study [41], we can conclude that three factors played a role in the toxicology of the mixtures: (1) the dosage; (2) the duration of exposure; (3) the compounds’ interaction with each other and with the cell.

Overall increased time of exposure increased the likelihood of the toxic effect to the cell, regardless of the mixture composition or the cell type. Dosage was chemical-dependent; while metals seem to have a straightforward dose-dependent toxicity, NPs have the additional aggregation phenomena that must be considered both in single and mixture exposure. As such, cytotoxicity of mixtures can be either more pronounced or attenuated than when cells are exposed to individual compounds. Co-exposure can result in:

1. potentiation effects: if aggregates are of small size and can be easily uptaken by the cell, if adsorption to NP facilitates the entrance of metals, and/or by the toxicity of the metal alone;
2. antagonism effects: if the metals cause the formation of large NP aggregates that hinder their uptake by the cells, block the other contaminants’ access to the cell, and/or if the NP (e.g., CeO_2_NPs) have the ability to act as antioxidant and reduce oxidative stress in the cells.

Our study proves that when addressing NP and metal(oid) co-exposure effects it it is particularly important to: (a) understand the physical-chemical consequences resulting from the contaminants’ simultaneous presence in the exposure media and how it can affect their availability and toxicity to the cell in that media; and (b) address the chronic effects, especially if meta(loid)ls with very long half-life in humans are present in the mixture, as our data shows that prolonged exposure resulted in significant inhibition of cell proliferation even at low concentration of the contaminants. Therefore, although it represents an experimental and observational challenge, understanding how mixtures affect the well-being of the population is critical to decide on health-related strategies and policies designed to prevent health loss in a population increasingly exposed to multiple contaminants.

## Data Availability

The data presented in this study are available in the tables, figures and Supplementary Materials of this article.

## Supplementary Materials

The following are available online at https://www.mdpi.com/article/10.3390/ijms23052737/s1.
